# Feasibility and Cardiometabolic Effects of Time-Restricted Eating in Patients with Metabolic Syndrome

**DOI:** 10.3390/nu16121802

**Published:** 2024-06-07

**Authors:** Iwona Świątkiewicz, Jarosław Nuszkiewicz, Joanna Wróblewska, Małgorzata Nartowicz, Kamil Sokołowski, Paweł Sutkowy, Paweł Rajewski, Krzysztof Buczkowski, Małgorzata Chudzińska, Emily N. C. Manoogian, Pam R. Taub, Alina Woźniak

**Affiliations:** 1 Division of Cardiovascular Medicine, University of California San Diego, La Jolla, CA 92037, USA; ptaub@health.ucsd.edu; 2 Department of Medical Biology and Biochemistry, Collegium Medicum, Nicolaus Copernicus University, 85-092 Bydgoszcz, Poland; jnuszkiewicz@cm.umk.pl (J.N.); jwroblewskapraca@gmail.com (J.W.); sokolowskikamil@tlen.pl (K.S.); p.sutkowy@cm.umk.pl (P.S.); al1103@cm.umk.pl (A.W.); 3 Clinical Nutrition Team, Oncology Center—Professor Franciszek Łukaszczyk Memorial Hospital, 85-796 Bydgoszcz, Poland; nartowiczgosia@gmail.com; 4 Center for Obesity and Metabolic Disorders Treatment, 85-676 Bydgoszcz, Poland; rajson@wp.pl; 5 Faculty of Health Sciences, University of Health Sciences in Bydgoszcz, 85-067 Bydgoszcz, Poland; 6 Department of Family Medicine, Collegium Medicum, Nicolaus Copernicus University, 85-094 Bydgoszcz, Poland; buczkowskik@cm.umk.pl; 7 Department of Nutrition and Dietetics, Collegium Medicum, Nicolaus Copernicus University, 85-626 Bydgoszcz, Poland; malgorzata.chudzinska1@wp.pl; 8 Regulatory Biology Laboratory, Salk Institute for Biological Studies, La Jolla, CA 92037, USA; emanoogian@salk.edu

**Keywords:** time-restricted eating, metabolic syndrome, abdominal obesity, body weight, elevated blood pressure, impaired glucose metabolism, cardiometabolic risks, circadian rhythm, eating window, m-health applications

## Abstract

Metabolic syndrome (MetS) and a prolonged daily eating window (EW) are associated with circadian rhythm disruption and increased cardiometabolic risk. Misalignment between circadian timing system and daily rhythms of food intake adversely impacts metabolic regulatory mechanisms and cardiovascular function. Restricting the daily EW by imposing an eating–fasting cycle through time-restricted eating (TRE) can restore robust circadian rhythms, support cellular metabolism, and improve cardiometabolic health. The aim of this study was to assess a feasibility of 12-week TRE intervention with self-selected 10 h EW and effects of TRE on EW duration, cardiometabolic outcomes, daily rhythms of behavior, and wellbeing in Polish patients with MetS and EW ≥ 14 h/day. Dietary intake was monitored with a validated myCircadianClock application (mCC app). Adherence to TRE defined as the proportion of days recorded with mCC app in which participants satisfied 10-h TRE was the primary outcome. A total of 26 patients (aged 45 ± 13 years, 62% women, 3.3 ± 0.5 MetS criteria, EW 14 ± 1.5 h/day) were enrolled. Coexistence of increased waist circumference (WC) (96% of patients), elevated fasting plasma glucose (FPG) (77%), and elevated blood pressure (BP) (69%) was the most common MetS pattern (50%). TRE intervention (mean duration of 81.6 ± 12.6 days) led to reducing daily EW by 28% (*p* < 0.0001). Adherence to TRE was 87 ± 13%. Adherence to logging food intake on mCC app during TRE was 70 ± 27%. Post TRE, a decrease in body weight (2%, 1.7 ± 3.6 kg, *p* = 0.026), body mass index (BMI) (1%, 0.5 ± 1.2 kg/m^2^, *p* = 0.027), WC (2%, 2.5 ± 3.9 cm, *p* = 0.003), systolic BP (4%, 4.8 ± 9.0 mmHg, *p* = 0.012), FPG (4%, 3.8 ± 6.9 mg/dL, *p* = 0.037), glycated hemoglobin (4%, 0.2 ± 0.4%, *p* = 0.011), mean fasting glucose level from continuous glucose monitor (CGM) (4%, 4.0 ± 6.1 mg/dL, *p* = 0.002), and sleepiness score (25%, 1.9 ± 3.2 points, *p* = 0043) were observed. A significant decrease in body weight (2%), BMI (2%), WC (3%), mean CGM fasting glucose (6%), sleepiness score (27%), and depression score (60%) was found in patients with mean post-TRE EW ≤ 10 h/day (58% of total), and not in patients with EW > 10 h/day. Adherence to TRE was higher in patients with post-TRE EW ≤ 10 h/day vs. patients with EW > 10 h/day (94 ± 6% vs. 77 ± 14%, *p* = 0.003). Our findings indicate that 10-h TRE was feasible in the European MetS population. TRE resulted in reducing daily EW and improved cardiometabolic outcomes and wellbeing in patients with MetS and prolonged EW. Use of the mCC app can aid in implementing TRE. This pilot clinical trial provides exploratory data that are a basis for a large-scale randomized controlled trial to determine the efficacy and sustainability of TRE for reducing cardiometabolic risks in MetS populations. Further research is needed to investigate the mechanisms of TRE effects, including its impact on circadian rhythm disruption.

## 1. Introduction

Metabolic syndrome (MetS) occurs in approximately 25–35% of adults and is associated with an increased cardiometabolic risk [1,2,3,4,5,6,7,8,9,10]. Available evidence indicates that prevalence of MetS has been increasing [10]. Based on data from NHANES 1999 to 2018 and using a harmonized definition of MetS, the overall MetS prevalence increased over this period from 36% to 47% [10]. MetS doubles the long-term risk of developing cardiovascular disease (CVD) and is associated with a fivefold increase in the risk of type 2 diabetes (T2D) [2,3,11]. The increasing prevalence of MetS is linked to a common occurrence of cardiometabolic risk factors such as excess weight, abdominal obesity, high systolic blood pressure (BP), elevated glucose levels, dyslipidemia, low physical activity, excessive caloric intake, poor diet quality, and the aging population [1,10,12,13,14,15,16]. Notably, about 65% of obese individuals satisfied the criteria for MetS [5]. Obesity is a global epidemic that has been continuously growing and more than doubled in most countries from 1990 to 2022 [1,10,15,16]. Comparing NHANES data from 1999 to 2000 with data from 2017 to 2018 shows that the prevalence of obesity increased from 28% to 43% among US males and from 33% to 42% among US females [10]. In addition, elevated fasting plasma glucose (FPG) and high body mass index (BMI) are among the leading risk factors that displayed the largest increases in risk exposure over the last three decades [15]. Identifying and addressing risk factors can reduce cardiometabolic risks; however, the efficacy of currently used preventive and therapeutic strategies based on calorie restriction and increased physical activity is insufficient [4,17,18,19,20,21,22,23].

Misalignment between circadian timing system and daily rhythms of food intake adversely impacts metabolic regulatory mechanisms and cardiovascular function [24,25,26]. Circadian rhythm disruption that may be caused by erratic eating patterns such as a prolonged daily eating window (EW) is associated with an increased risk of cardiometabolic disorders such as obesity, MetS, T2D, and CVD [27,28,29,30,31,32,33,34]. In the majority of people, the daily period of dietary intake is at least 15 h or greater, and only ~10% of adults habitually maintain fasting of >12 h/day [35,36,37,38]. Time-restricted eating (TRE) is a lifestyle intervention in which eating is restricted to a reduced, fixed number of hours per day, which supports an adequate fasting period [24,39]. A number of experimental studies indicated that maintaining an appropriate daily rhythm of eating–fasting cycles through TRE sustains robust circadian rhythms, which improves cellular metabolism and supports metabolic homeostasis and cardiovascular function [24,25,26]. TRE can restore normal levels and/or normal daily rhythms in several mRNAs, proteins, and metabolites that are implicated in metabolic homeostasis of glucose, lipids, redox, and mitochondria function, and regulates circulating adiponectin and leptin levels [24,25,26]. Several small-scale clinical studies employing TRE, which were conducted mostly in US populations with metabolic disorders, provided encouraging results in terms of cardiometabolic benefits such as body weight (BW) loss and a decrease in EW duration, fat mass, and energy intake, as well as improved glucose tolerance, insulin resistance, glycemic control, atherogenic lipid levels, self-reported sleep, and reduced BP [25,26,37,38,40,41,42,43,44,45,46,47,48,49,50,51,52,53,54]. In previous TRE studies, EW was self-selected or imposed by study protocol and varied from 4 to 12 h with the start and end time of EW indicating an “early TRE” pattern (eating early in the day) or “late TRE” pattern (with a phase delay to late hours in the day) [25,26,37,38,40,41,42,43,44,45,46,47,48,49,50,51,52,53,54]. However, data on a feasibility and cardiometabolic effects of TRE in European populations with metabolic disorders including subjects with MetS components are limited [25,40,50]. Moreover, while the results of TRE were investigated in a small-scale study of US patients with MetS [38], cardiometabolic effects of TRE in European MetS populations have not been specifically addressed in previous studies.

The aim of the TREMNIOS (Time-Restricted Eating on Metabolic and Neuroendocrine homeostasis, Inflammation, and Oxidative Stress) pilot clinical trial is to evaluate a feasibility of TRE intervention and collect exploratory data assessing changes in cardiometabolic outcomes, daily rhythms of behavior, and wellbeing in Polish patients with MetS and a prolonged daily EW. The underlying hypothesis is that restricting a daily EW by imposing an eating–fasting cycle through TRE without a predefined change in nutrition quality and quantity or the intensity of physical activity will restore robust circadian rhythms, support metabolic regulatory mechanisms and cardiovascular function, and improve cardiometabolic outcomes and overall health of patients with MetS.

## 2. Materials and Methods

### 2.1. Study Design and Participants

**Figure 1:**
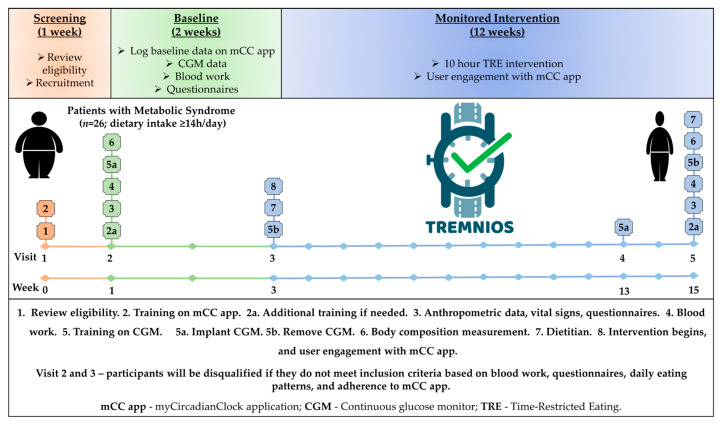
Study design.

TREMNIOS is a multicenter prospective single-arm pilot clinical trial with a pre–post intervention design that was performed in a Polish population of adult patients with MetS and an eating period of ≥14 h per day. Details on the trial protocol were described previously [55]. The essential components of the study design and its timeline are depicted in Figure 1.

Participants were recruited from the clinics at the Collegium Medicum, Nicolaus Copernicus University, Bydgoszcz, Poland (CM), and at the Center for Obesity and Metabolic Disorders Treatment, Bydgoszcz, Poland. This study consisted of 1 week of screening, 2 weeks of baseline assessment, and 12 weeks of monitored TRE intervention. During this study, patients underwent 5 visits.

At Visit 1 (Week 0—screening), eligibility criteria were verified based on a detailed interview and standard physical examination performed by a physician from the research team. Visit 1 also entailed an introduction to a custom-made smartphone application, i.e., the myCircadianClock application (mCC app), that was used throughout the study to assess eating (dietary timing and intake, and compliance with TRE) and sleep pattern [37].

Participants had to satisfy three or more out of five criteria of MetS diagnosis according to the International Diabetes Federation (IDF) recommendations [4], self-reported dietary intake of ≥14 h/day, regular daytime schedule of activity, self-reported habitual sleep duration of >6.5 h, and a smartphone with an Apple operating system (OS) or Android OS. The exclusion criteria included diagnosis of diabetes mellitus, shift work, recent history of major adverse cardiac events, other active or uncontrolled medical conditions, history of eating disorder and bariatric surgery, participation in the weight management program, special or prescribed diet for other reasons, substance abuse, depression, sleep apnea, and treatment with antidepressants, medication affecting glucose metabolism or appetite, or immunosuppression. The eligibility criteria are listed in Table 1.

At Visit 2 (Week 1—baseline), anthropometric data and vital signs including heart rate (HR) and BP were collected. Visit 2 also entailed a fasting blood work and training on the mCC app. Participants were asked to log their habitual dietary intake and timing of sleep on the mCC app for 2 weeks. They also had continuous glucose monitor (CGM) to continuously record blood glucose for 2 weeks and underwent body weight and composition measurement. Participants also completed health questionnaires.

At Visit 3 (Week 3—intervention begins), participants returned to remove the CGM and review their mCC app data. Participants who were eligible based on blood work results and logging on the mCC app (recording a minimum of two caloric entries > 5 h apart for a given day for at least 5 days a week and having an eating interval of ≥14 h/day) entered a 12-week monitored TRE intervention. They met with a dietitian in person for behavioral nutritional counseling. Participants were asked to restrict their food intake to 10 h a day within a self-selected EW and log their meals and sleep timing on the mCC app. During monitored intervention, all participants received education and support, including daily prompts from the mCC app.

At Visit 4 (Week 13), which is 10 weeks into the intervention period, participants returned to the clinic to have the CGM device placed for 2 weeks. At Visit 5 (Week 15—intervention ends), participants visited the clinic to remove the CGM. On this visit, they also had their fasting blood work analyzed, vital signs taken, and body weight and composition measured, and were asked to complete the same questionnaires as during Visit 2.

This study was conducted in accordance with the Declaration of Helsinki. Approval from the Bioethics Committee of the Nicolaus Copernicus University in Toruń, Collegium Medicum in Bydgoszcz, Poland was obtained (KB 107/2019). All participants provided informed consent. Approval from the Bioethics Committee of the Salk Institute for Biological Studies, La Jolla, USA was obtained for the use of the mCC app data (IRB 15-0003/2019). All participants received information about the terms and conditions of the use of mCC app before providing informed consent. This study was registered at www.clinicaltrials.gov (ClinicalTrials.gov, ID: NCT04328233, registered on 30 March 2020).

Data were collected by researchers with training and experience in clinical assessment and data management. The data are stored with the highest possible level of security. Completed data forms or other hard-copy documents containing protected health information are kept in a locked file at the Department of Medical Biology and Biochemistry, CM. Data were entered into an electronic deidentified database by authorized team members; participants were identified only using a unique number. The electronic data are stored on a secure server. The principal investigator (I.Ś.) and designated team members review data collection forms on an ongoing basis for data completeness and accuracy, and protocol compliance. Access to data with identifiers is restricted to authorized team members and regulatory authorities.

### 2.2. TRE Intervention

The TRE intervention was based on restricting daily food intake to 10 h a day with fasting for 14 h. Participants started the intervention by selecting a 10-h EW (between 7 a.m. and 9 p.m.) that best suited their lifestyle based on his/her baseline eating pattern from the first 2 weeks of baseline assessment by the mCC app. Participants were asked to consume all meals within a chosen EW that was entered in the mCC app. The last meal (including non-water beverage) had to be consumed at least 2 h prior to the typical bedtime. Beverages that include caffeine or artificial sweeteners were not allowed outside of the 10-h EW. TRE was the only intervention, and participants were not instructed to change their habits regarding physical activity or the quality, quantity, or caloric content of their diet.

Other details on TRE intervention were described in the protocol of the TREMNIOS study [55].

### 2.3. myCircadianClock Application

The mCC app is a free validated smartphone application that was developed at the Salk Institute for Biological Studies, La Jolla, USA [37]. The mCC app is designed to run on Android and iOS devices and uses an HIPAA-compliant Amazon Web Server for server-side operations. For the TREMNIOS clinical trial, the mCC app was customized to be used in a single group assignment (TRE intervention group) study.

During the baseline period, data from the mCC app were used to evaluate habitual food intake, adherence to logging on the app, and sleep duration. During the monitored TRE intervention, adherence to TRE, adherence to logging on the mCC app, EW duration and timing, and sleep duration were evaluated. After self-selecting a 10-h EW, a chosen EW was highlighted in the app so that participants could visualize food intake within their set interval. Participants were asked to log all food intake and sleep every day for the baseline period and for the duration of TRE intervention between Visits 3 and 5 (Figure 1). With the mCC app, logging food intake is possible by using the picture feature or entering the name of food and the approximate time that food was consumed. Because a Polish version of mCC app is not available, we provided participants with the list of common food with the names both in Polish and English.

**Figure 2:**
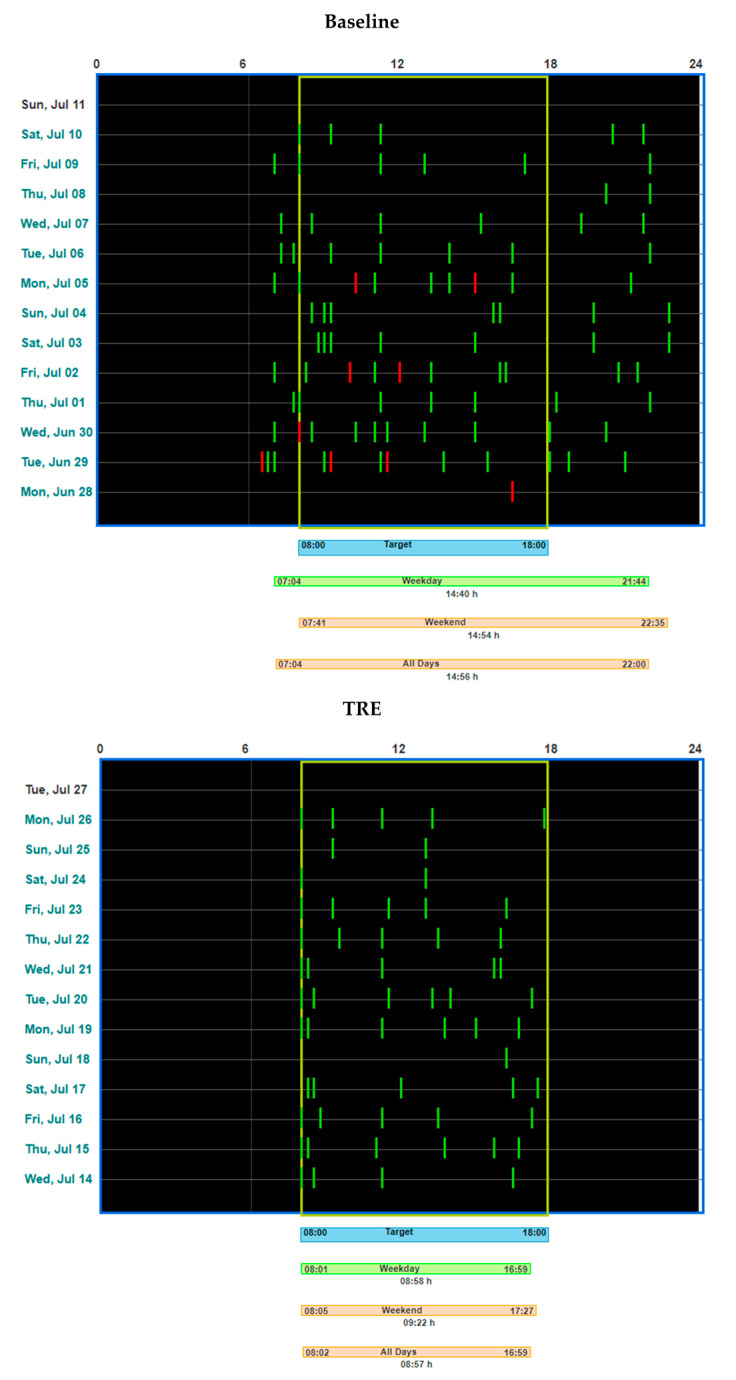
“Feedogram” raster plot obtained by the myCircadianClock application in an example study participant during baseline period (the upper part of figure) and during time-restricted (TRE) intervention (the lower part of figure).

The designated members of research team had password-protected access to the real-time data on participants’ daily logs, which were displayed on a study dashboard of the app server-side. This approach allowed for monitoring food intake data, performing real-time tracking of participants’ adherence to TRE and logging, and following up on participants with inconsistent logging as needed. Adherence to logging on the mCC app was determined by a minimum of two caloric entries > 5 h apart for a given day. Adherence to the designated EW implies that ~95% caloric items were contained within a 15 min buffer on each side of the self-selected 10-h EW. The “feedogram” raster plot offers a visual summary of the participant’s eating pattern (Figure 2).

The proportion of the total number of days recorded with the mCC app during the monitored TRE intervention period in which participants satisfied a requirement of a 10-h EW was used to quantify the adherence to the TRE intervention. This proportion is considered as the primary outcome measure. Change in mean daily EW duration—defined as the duration from the first to last caloric intake over the 24 h cycle collected via the mCC app—between 2-week baseline period and 12-week monitored TRE intervention was calculated. Also, change in sleep duration collected via the mCC app between the baseline period and TRE intervention period was calculated.

Other details on the use of the mCC app are included in the protocol of the TREMNIOS study [55].

### 2.4. Continuous Glucose Monitor

The Abbott Freestyle Libre Pro CGM was used for measurement of interstitial fluid glucose every 15 min for 14 full days, using a subcutaneous sensor placed in the upper arm area. The participant had the CGM for 2 weeks during the baseline period and at the last 2 weeks of 12-week TRE intervention. Participants were fitted with a CGM and instructed on its use. They were blinded to their CGM data during the entire study.

Changes in mean fasting glucose levels and mean daily glucose levels obtained by CGM between 2-week baseline period and the last 2 weeks of 12-week TRE intervention were calculated.

### 2.5. Anthropometry, Body Composition, Cardiovascular Parameters, Questionnaires, and Blood Samples

The anthropometric measurements were performed at Visits 2 and 5. Participants had their BW and height measured in the fasted state on a digital scale. The accuracy of the measurements is 0.1 kg and 0.5 cm, respectively. BMI was calculated using the formula [weight(kg)/height^2^(m^2^)], and the cut-off points of the World Health Organization (WHO) were used. Waist circumference (WC) was measured in a fasted state immediately above the iliac crest using an anthropometric tape accurate to 0.5 cm. Changes in BW, BMI, and WC between Visits 2 and 5 were calculated.

The body composition measurements were performed at Visits 2 and 5 using bioelectrical impedance technology with the Tanita Scale DC 430U (Tokyo, Japan). Body fat percentage (BF), visceral fat rating (VF), and greater muscle mass were evaluated. Changes in body composition components between Visits 2 and 5 were calculated.

Systolic and diastolic BP and HR measurements were taken at Visits 2 and 5 under resting (after a 5 min rest) and fasting conditions, resulting in an output that was an average of three readings 1–2 min apart. Changes in systolic BP, diastolic BP, and HR between Visits 2 and 5 were calculated.

The Beck Depression Inventory-II (BDI-II) and the Epworth Sleepiness Scale (ESS) questionnaires, which were used to screen for depression (a BDI-II score of >29) and exclude sleep apnea (an ESS score of >16), respectively, were collected at Visits 2 and 5. The BDI-II was used for measuring depression severity, i.e., higher total scores indicate more severe depressive symptoms. The ESS was used to assess self-reported sleepiness. Changes in questionnaire scores between Visits 2 and 5 were calculated.

Participants had their blood drawn at Visits 2 and 5 in the morning after overnight fasting of 12 h. Measurements of routine laboratory tests such as a complete blood count (CBC), including hemoglobin, red blood count, leukocyte count and platelet count, FPG, creatinine, aspartate aminotransferase (AST), alanine aminotransferase (ALT), uric acid, thyroid-stimulating hormone (only at Visit 2), and glycated hemoglobin (HbA1c), and lipid profile, including total cholesterol (TC), low-density lipoprotein cholesterol (LDL-C), high-density lipoprotein cholesterol (HDL-C), non-high-density lipoprotein cholesterol (non-HDL-C), and triglycerides (TG), were taken in serum samples in a certified analytical laboratory. Changes in blood tests results between Visits 2 and 5 were calculated.

### 2.6. Dietetic Analysis

The food intake data (photo and/or annotation entries) were downloaded from the server side of the app and dietary analyses were performed by a registered dietitian to calculate the overall calorie intake. Three-day food records from the baseline period and the last twelve days of TRE intervention were randomly chosen and analyzed to characterize the macronutrient composition of the diet. Caloric estimates were made using the CalorieKing database [56]. Mean number of daily eating occasions was also evaluated. Changes in calorie intake and mean number of eating occasions/day assessed at Visits 3 and 5 were calculated. A 3-day dietary intake protocol is consistent with a protocol used by Wilkinson and Manoogian et al. [38] in the study of the US patients with MetS.

### 2.7. Primary and Secondary Outcomes Measures

The adherence to TRE intervention is the primary outcome defined as the proportion of the total number of days recorded with mCC app during the monitored TRE intervention period (i.e., between Visit 3 and Visit 5) in which participants satisfied a requirement of a 10-h EW [55].

The main secondary outcome measures include changes in BW and FPG between Visit 2 and Visit 5 [55]. Other secondary outcome measures include post-TRE changes in EW duration, systolic BP, diastolic BP, HR, BMI, WC, BF, VF, muscle mass, lipids and HbA1c levels, as well as mean 24 h glucose levels and mean fasting blood glucose levels obtained by CGM, calorie intake, sleep duration, health questionnaires scores, and adherence to logging on the mCC app [55].

### 2.8. Statistical Analysis

The study results were subjected to analysis of parametricity using the Kolmogorov–Smirnov test for normality and Levene’s test for homogeneity of variances. Differences between baseline and post-TRE study timepoints were analyzed with a dependent t-test in the case of parametric results, and with a Wilcoxon test in the case of nonparametric results. Differences between two subgroups depending on post-TRE EW (>10 h vs. ≤10 h) were compared using an independent *t*-test (parametric results) or Mann–Whitney U test (nonparametric results). Differences in post-TRE changes in EW duration and adherence to logging on mCC app between two subgroups depending on post-TRE EW (>10 h vs. ≤10 h) were analyzed using a post-hoc test (Tukey’s honestly significant difference (HSD) test for unequal N) in a one-way analysis of variance (ANOVA) (parametric results), or using post-hoc analysis (Dunn’s test) in a Kruskal–Wallis H test.

Qualitative variables related to baseline demographic and clinical characteristics were analyzed using detailed two-way tables with Fisher’s exact test (two-tailed *p*), excluding “Unemployed/Employed/Retired *n* (%)”, which was analyzed with the Pearson χ2 statistic. An additional analysis of the results was the linear regression analysis using Pearson’s correlation coefficient (r).

The adherence to TRE intervention expressed as the percentage of days with achievement of required 10-h EW during the TRE intervention monitored by mCC app was calculated per participant, and then reported as a group mean with standard deviation.

Differences were statistically significant in the given test at *p* < 0.05. The results are presented as arithmetic means and standard deviations.

## 3. Results

The study participants were screened for eligibility and recruited from the clinics from October 2019 to October 2023. The recruitment was temporarily deferred due to the COVID-19 pandemic from March 2020 to September 2020, from November 2020 to March 2021, and from December 2021 to February 2022. After confirming patient eligibility at Visit 1, 36 patients with the diagnosis of MetS and self-reported EW of ≥14 h were enrolled in this study. After enrollment, one patient resigned at Visit 2. Thus, 35 patients started the baseline period. At Visit 3, one patient resigned, and three patients were excluded for not meeting inclusion criteria (one patient due to the diagnosis of T2D and insufficient logging on the mCC app, and two patients due to insufficient logging on the mCC app). Thus, 31 patients began the TRE intervention. During the TRE phase, one patient was excluded at Visit 5 and four patients (13% of patients undergoing TRE) resigned from the participation in the study between Visit 3 and Visit 5 owing to difficulties in complying with the TRE requirements and use of the mCC app. Thus, 26 patients completed the 12-week monitored TRE intervention and were included in this analysis. Out of them, 15 patients (57.7% of total) achieved a goal of mean daily EW duration of ≤10 h during the TRE intervention. The majority of patients (24 of 26 patients, i.e., 92% of total) self-selected a 10-h EW with a start from 8 a.m. to 11 a.m. and an end at or after 6 p.m. (from 6 p.m. to 9 p.m.), which indicates that most study participants chose a “late TRE” pattern. No serious adverse events were reported by the participants throughout the TRE intervention.

### 3.1. Baseline Demographic and Clinical Characteristics

The baseline demographic and clinical characteristics for the whole study group of patients with MetS undergoing TRE intervention are displayed in Table 2.

The majority (62%) of the studied population (with a mean age of 45 years) were women. Mean EW duration recorded with the mCC app within the 2-week baseline period was 14.0 ± 1.5 h/day. Most patients (77%) were obese (with a mean BMI of 35 kg/m^2^). All patients satisfied at least three criteria (mean of 3.3 criteria/patient) out of five IDF criteria for the diagnosis of MetS [4]. Nine patients (35% of total) met four MetS criteria. Specifically, high WC (with a mean of 114 cm), elevated FPG (with a mean of 102 mg/dL), and elevated BP (with a mean of 132/85 mmHg) were found in the majority of patients, i.e., in 96%, 77%, and 69%, respectively [4]. Elevated fasting TG (with a mean of 154 mg/dL) and reduced HDL-C (with a mean of 53 mg/dL) were less common (54% and 35% of patients, respectively). Coexistence of high WC, elevated FPG, and elevated BP was the most common pattern of metabolic disorders characterizing MetS, which occurred in 13 patients (50% of total). Other MetS patterns occurred less frequently, e.g., the combination of high WC, elevated FPG, and elevated TG (six patients, 23% of total); combination of high WC, elevated BP, and elevated TG or reduced HDL-C (four patients, 15% of total). In addition, 10 patients (39% of total) had elevated baseline HbA1c of ≥5.7%. In total, 22 patients (85%) were diagnosed with glucose metabolism abnormalities because they had FPG ≥ 100 mg/dL and/or HbA1c of ≥5.7%.

According to the Systematic Coronary Risk Estimation2 (SCORE2) algorithm, the mean 10-year risk of fatal and non-fatal cardiovascular (CV) events (myocardial infarction, stroke) in the study participants was 4.7% (Table 2), which can be categorized as a high CV risk [23]. Moreover, the mean LDL-C was 117 mg/dL, which is a non-optimal LDL-C level according to the European Society of Cardiology (ESC) recommendations [23]. Specifically, 18 patients (69% of total) had an LDL-C of ≥100 mg/dL. Also, ALT level was mildly elevated (mean 37.3 U/L; the normal range 4-36 U/L).

Nine patients (35% of total) had prior diagnosis of mild systemic essential hypertension (HTN) and received antihypertensive treatment including angiotensin-converting enzyme inhibitor or angiotensin receptor blocker (eight patients), beta blocker (two patients), calcium channel antagonist (one patient), and non-potassium-sparing diuretic (two patients). Three patients (12% of total) took statins for prior diagnosis of hyperlipidemia. No dose adjustments of medications were made during the study period.

Family history of premature atherosclerotic CVD was reported by 46% of participants. The same percentage of the studied population reported regular aerobic exercise. Most patients were married, had higher education, were employed, and lived in the city.

The comparison of baseline demographic and clinical characteristics for two patient subgroups depending on mean TRE daily EW duration (>10 h or ≤10 h) is shown in Table 2. In this study, the subgroups of patients who had mean TRE daily EW > 10 h and EW ≤ 10 h are referred to as subgroups “EW > 10” and “EW ≤ 10”, respectively. At baseline, no differences were observed between subgroups “EW > 10” and “EW ≤ 10” in age, gender, EW duration, adherence to logging on the mCC app, demographic and clinical characteristics, or cardiometabolic and biochemical parameters. The most common MetS pattern was similar in the “EW ≤ 10” and “EW > 10” subgroups and comprised increased WC (100% vs. 91% of patients, respectively), elevated FPG (80% vs. 73%), and elevated BP (73% vs. 64%).

### 3.2. TRE Intervention: Duration, Adherence to TRE, and Adherence to Logging on mCC App

The 12-week TRE intervention was conducted between Visit 3 and Visit 5. The mean TRE intervention duration was 81.6 ± 12.6 days (Table 3). The mean adherence to TRE intervention and mean adherence to logging on the mCC app during TRE intervention was 87 ± 13.2% and 70 ± 27%, respectively (Table 3).

### 3.3. TRE Intervention: Changes in Eating Window Duration, Adherence to Logging on the mCC App, Cardiometabolic Outcomes, and Wellbeing Outcomes

#### 3.3.1. TRE Intervention: Changes in EW Duration, Adherence to Logging on the mCC App, Cardiometabolic Outcomes, and Wellbeing Outcomes for the Whole Study Group

Changes in EW duration, logging on the mCC app, cardiometabolic outcomes, and ESS and BDI-II scores between baseline period and TRE intervention for the whole study group of patients with MetS undergoing TRE are displayed in Table 4.

**Figure 3:**
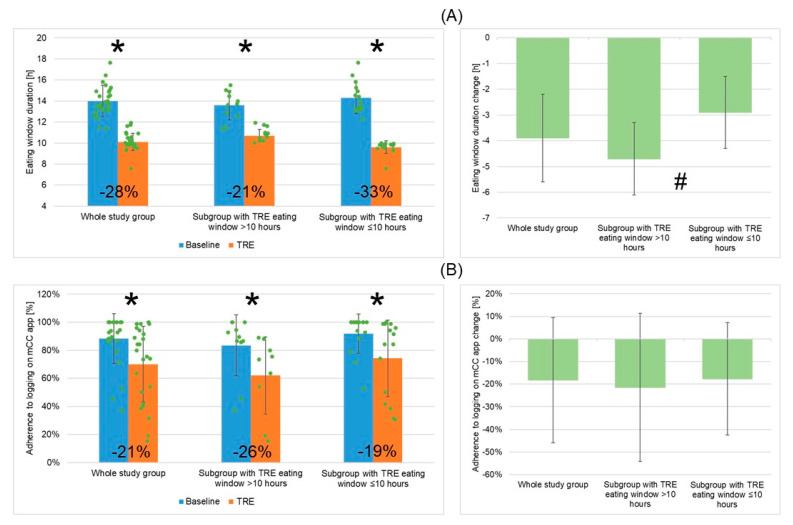
Changes in mean daily eating window duration (**A**) and adherence to logging on mCC app (**B**) for the whole study group of patients with metabolic syndrome undergoing TRE and two patient subgroups depending on mean TRE daily eating window duration (>10 h or ≤10 h) within groups (left panels) and between groups (right panels). The left panels include data points for individual study participants and the percentage change in mean values between the baseline and TRE intervention periods are shown. Abbreviations: mCC app—myCircadianClock application; TRE—time-restricted eating; *—*p* < 0.05; #—0.05 ≥ *p* < 0.1.

Based on the mCC app logs, mean daily EW duration during the 12-week TRE intervention was significantly reduced (by an average of 28%, i.e., ~3.9 h), from 14.0 ± 1.5 h to 10.1 ± 0.8 h (Figure 2, Figure 3A left panel).

Mean adherence to logging on the mCC app decreased significantly during TRE intervention compared to baseline period (70% vs. 88%; Figure 3B left panel).

**Figure 4:**
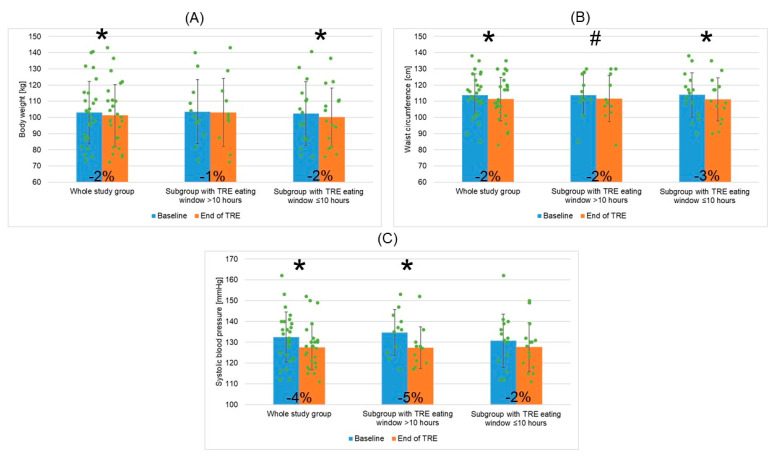
Changes in body weight (**A**), waist circumference (**B**), and systolic blood pressure (**C**) for the whole study group of patients with metabolic syndrome undergoing TRE and two patient subgroups depending on mean TRE daily eating window duration (>10 h or ≤10 h). Data points for individual study participants and the percentage change in mean values between the baseline and the end of TRE intervention are shown. Abbreviations: TRE—time-restricted eating; *—*p* < 0.05; #—0.05 ≥ *p* < 0.1.

Post TRE intervention, BW and BMI decreased significantly (by ~2%, i.e., ~1.7 kg and by ~1.4%, i.e., ~0.5 kg/m^2^; respectively, Figure 4A). Also, a significant decrease in WC and hip circumference was found (by ~2%, i.e., ~2.5 cm; Figure 4B, and by ~2%, i.e., 2.1 cm, respectively). BF, VF, and muscle mass did not change post TRE. ALT decreased significantly by ~20%.

A significant decrease in systolic BP (by ~4%, i.e., ~4.8 mmHg), and not diastolic BP or HR, was observed post TRE (Figure 4C). Post-TRE mean systolic BP reached optimal level according to the ESC recommendations for individuals with high CV 50 years of age or younger [23]. Also, the SCORE2 value decreased significantly post TRE.

**Figure 5:**
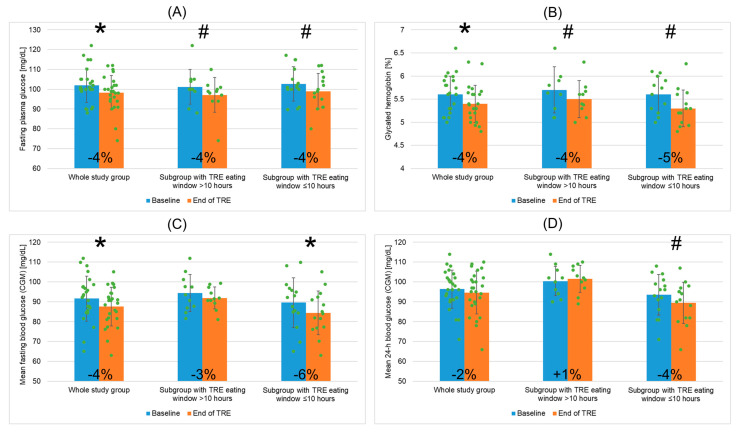
Changes in fasting plasma glucose (**A**), glycated hemoglobin (**B**), mean fasting blood glucose obtained by CGM (**C**), and mean 24 h blood glucose obtained by CGM (**D**) for the whole study group of patients with metabolic syndrome undergoing TRE and two patient subgroups depending on mean TRE daily eating window duration (>10 h or ≤10 h). Data points for individual study participants and the percentage change in mean values between the baseline and the end of TRE intervention are shown. Abbreviations: CGM—continuous glucose monitor; TRE—time-restricted eating; *—*p* < 0.05; #—0.05 ≥ *p* < 0.1.

Regarding glycemic parameters, FPG (Figure 5A) and HbA1c (Figure 5B) decreased significantly post TRE (by ~3.7%, i.e., ~3.8 mg/dL, and by ~3.6%, i.e., ~0.2%, respectively). Post TRE, mean FPG was <100 mg/dL. Also, a significant decrease in mean CGM fasting blood glucose level was recorded at the end of TRE intervention (by ~4.4%, i.e., ~4.0 mg/dL, Figure 5C). In addition, mean CGM 24 h blood glucose level had a tendency to decrease post-TRE (Figure 5D). Post-TRE, the mean CGM fasting and mean CGM 24-h blood glucose levels correlated with the mean TRE daily EW duration (r = 0.43, *p* = 0.045 and r = 0.44, *p* = 0.038, respectively).

**Figure 6:**
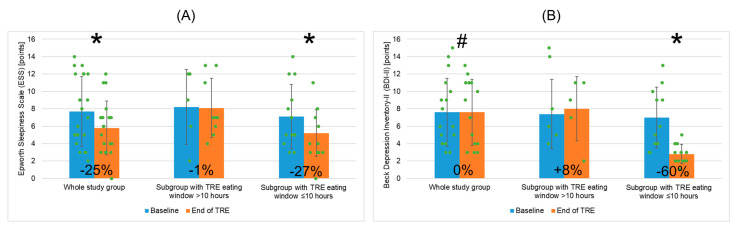
Changes in Epworth Sleepiness Scale (**A**) and Beck Depression Inventory-II (**B**) for the whole study group of patients with metabolic syndrome undergoing TRE and two patient subgroups depending on mean TRE daily eating window duration (>10 h or ≤10 h). Data points for individual study participants and the percentage change in mean values between the baseline and the end of TRE intervention are shown. Abbreviations: BDI-II—Beck Depression Inventory-II; ESS—Epworth Sleepiness Scale; TRE—time-restricted eating; *—*p* < 0.05; #—0.05 ≥ *p* < 0.1.

Regarding the health questionnaires, the ESS score decreased significantly post TRE (by ~24.7%, i.e., ~1.9 points, Figure 6A) and the BDI-II score showed the tendency to decrease post TRE (Figure 6B).

No significant changes were observed post TRE in lipid levels, other biochemical parameters, and mean sleep duration. Mean daily caloric intake decreased significantly by ~16.1% post TRE. Also, mean number of eating occasions per day (based on the mCC app logs) was likely to decrease during the TRE intervention compared to baseline period.

#### 3.3.2. Adherence to TRE Intervention and Changes in EW Duration, Adherence to Logging on mCC App, Cardiometabolic Outcomes, and Wellbeing Outcomes for Two Patient Subgroups Depending on Mean TRE Daily EW Duration (>10 h or ≤10 h)

Adherence to TRE and changes in the analyzed outcomes post TRE intervention for two patient subgroups depending on mean TRE daily EW (i.e., subgroups “EW > 10” and “EW ≤ 10”) are shown in Table 3 and Table 5.

The adherence to TRE was significantly higher in patients from the subgroup “EW ≤ 10” compared to the subgroup “EW > 10” (94% vs. 77%) (Table 3). Mean adherence to logging on the mCC app decreased significantly during TRE in both subgroups (Figure 3B) and was lower post TRE in the subgroup “EW > 10” (62%) than in “EW ≤ 10” (74%).

Mean daily EW was reduced significantly during TRE in both subgroups (Figure 3A left panel), but more for “EW ≤ 10” (Figure 3A right panel). Specifically, mean daily EW was reduced by ~33%, i.e., ~4.7 h (to ~9.6 h) in “EW ≤ 10” and by ~21%, i.e., ~2.9 h (to 10.7 h) in “EW > 10”, and was significantly shorter during TRE in the subgroup “EW ≤ 10” than in “EW > 10”.

Post TRE, BW (Figure 4A), BMI, and WC (Figure 4B) decreased significantly only in the subgroup “EW ≤ 10”, and not in “EW > 10”. In “EW ≤ 10”, BW, BMI, and WC decreased by ~2.4 kg (~2.3%), ~0.8 kg/m^2^ (~2.3%), and ~2.8 cm (~2.5%), respectively.

Systolic BP decreased significantly post TRE only in the subgroup “EW > 10”; however, post-TRE systolic BP reached optimal mean value in both subgroups (Figure 4C).

Mean FPG (Figure 5A) and HbA1c (Figure 5B) showed a tendency to decrease post TRE in both subgroups. Also, a significant decrease in mean CGM fasting blood glucose level was observed at the end of TRE intervention in the subgroup “EW ≤ 10” (by ~6%, i.e., ~5.2 mg/dL), and not in “EW > 10” (Figure 5C). Also, mean CGM 24-h blood glucose level was more likely to decrease at the end of TRE intervention in “EW ≤ 10”, and not in “EW > 10” (Figure 5D). Post TRE, mean CGM 24-h blood glucose level was significantly lower in “EW ≤ 10” than in “EW > 10”.

While the lipid levels did not change in the subgroup “EW ≤ 10”, in “EW > 10”, TC was significantly reduced, and LDL-C and non-HDL-C were likely to decrease post TRE. Also, a significant decrease in AST level was found in “EW ≤ 10”. No significant changes were observed post TRE in other biochemical parameters and mean sleep duration in the studied subgroups.

The ESS and BDI-II scores decreased significantly post TRE only in the subgroup “EW ≤ 10” (by 27% and 60%, respectively) and post-TRE scores values were significantly lower in the subgroup “EW ≤ 10” than in “EW > 10”.

Mean daily caloric intake significantly decreased during TRE intervention in the subgroup “EW > 10 and was likely to decrease in the subgroup “EW ≤ 10”. The mean number of eating occasions per day significantly decreased during TRE in “EW ≤ 10”.

### 3.4. Primary and Main Exploratory Study Outcomes

The primary outcome—i.e., the adherence to the TRE intervention, defined as the proportion of the total number of days recorded with mCC app during the monitored TRE intervention in which participants satisfied a requirement of a 10 h EW—was 87 ± 13.2% (Table 3). The main secondary exploratory outcomes including post-TRE changes in BW and FPG comprise a decrease in BW (by 1.6%, i.e., 1.7 ± 3.6 kg, *p* = 0.026) and a decrease in FPG (by 3.7%, i.e., 3.8 ± 6.9 mg/dL, *p* = 0.037) (Table 4).

## 4. Discussion

Our findings support the usefulness of TRE as a lifestyle intervention for reducing cardiometabolic risks in patients with MetS and a prolonged daily EW. The 12-week TRE with a self-selected 10-h EW (mostly “late TRE” pattern) was feasible and led to improvements in cardiometabolic outcomes and wellbeing in the European MetS population. The high adherence to TRE, low dropout rate, and no reported serious adverse events in our study indicate that TRE can be sustainable preventative strategy in patients with MetS. Restricting daily EW from ≥14 h to ≤10 h in MetS patients resulted in reducing excess BW and abdominal obesity, elevated systolic BP and glucose levels, as well as improved daily eating patterns and wellbeing. We demonstrated that TRE was feasible and beneficial lifestyle intervention in middle-aged individuals with the diagnosis of MetS and high CV risk related to the presence of numerous cardiometabolic risk factors. A unique attribute of our study is the focus on evaluating the feasibility and effectiveness of TRE intervention in subjects with MetS in real-world clinical practice. Rigorous methodology including well-defined eligibility criteria and study outcomes, as well as recording daily food intake with the validated mCC smartphone app contribute to the strength of our study. To our knowledge, this is the first report that provides exploratory data on the feasibility and cardiometabolic effects of TRE intervention in the European population with the diagnosis of MetS and prolonged daily EW. The results of this pilot clinical trial can be used as a basis for a planned large-scale randomized controlled clinical trial.

The main findings of the TREMNIOS pilot clinical trial including the European population of middle-aged individuals with MetS and prolonged daily EW indicate that (i) coexistence of abdominal obesity, elevated FPG, and elevated BP was the most common MetS pattern (50% of study group); (ii) the most frequent cardiometabolic disorders in the studied MetS population included abdominal obesity with a high WC (96% of study group), obesity with a high BMI (77%), elevated FPG (77%), elevated BP (69%), elevated LDL-C (69%), elevated TG (54%), high HbA1c (39%), and reduced HDL-C (35%); (iii) the 10-year risk of CV events in the studied MetS population was high (the mean SCORE2 was 4.7%) despite a low prevalence of established CVD and exclusion of patients with T2D; (iv) 12-week TRE led to a significant reduction in mean daily EW (by ~28%, i.e., ~3.9 h); (v) the feasibility of the 12-week TRE intervention with a self-selected 10-h EW (mostly “late TRE” pattern) was satisfactory as evidenced by the mean adherence to TRE of 87%; (vi) 12-week TRE with a 10-h EW resulted in significant improvements in several cardiometabolic outcomes such as a decrease in BW (by ~2%), BMI (by ~2%), WC (by ~2%), hip circumference (by ~2%), systolic BP (by ~4%), FPG (by ~4%), HbA1c (by ~5%), and mean CGM fasting blood glucose level (~5%), as well as a favorable impact on liver function markers and self-reported sleepiness; (vii) a significant decrease in BW (by ~2%), BMI (by ~2%), WC (by ~3%), and mean CGM fasting blood glucose levels (by ~6%), as well as sleepiness score (by ~27%) and depression score (by ~60%) was observed in individuals who achieved EW ≤ 10 h/day during TRE (58% of patients); however, TRE effects were smaller or not observed in those with TRE EW > 10 h/day; (viii) adherence to TRE intervention was higher in individuals with TRE EW ≤ 10 h/day (94%) than in those with TRE EW > 10 h/day (77%).

According to the data on national CV mortality rates published by the WHO, Poland belongs to the cluster of countries of high CV risk [23]. In the studied MetS population characterized by multiple cardiometabolic risk factors, an individual’s 10-year risk of fatal and non-fatal CV events was estimated to be as high as ~4.7% [23]. In addition, an erratic daily eating pattern with prolonged EW of ≥14 h/day may be associated with an increased cardiometabolic risk [31,36,37,38,43]. Notably, this high CV risk refers to apparently healthy middle-aged people with a low prevalence of established CVD (i.e., well-controlled mild HTN in 35% of patients) and a lack of the diagnosis of T2D. It should also be noted that cardiometabolic risk factors were neither recognized nor addressed in most study participants before enrollment in this study. While 46% of study participants reported regular aerobic exercise, no other specific lifestyle modifications targeting excess weight, abdominal obesity, and non-optimal LDL-C, glucose, and BP levels were implemented before enrollment in this study. Regarding pharmacotherapy, only 35% of this MetS population received antihypertensive treatment and 12% took statins despite the presence of elevated BP and LDL-C in the majority of participants (69%). Compared to the US population of 19 subjects with MetS undergoing a 12-week TRE intervention [38], the European MetS population examined in our study was characterized by younger age (mean of 45 years vs. 59 years); greater percentage of women (62% vs. 32%) and white race individuals (100% vs. 63%); tendency to higher mean BW (103 kg vs. 98 kg), BMI (35 kg/m^2^ vs. 33 kg/m^2^), WC (114 cm vs. 109 cm), and BP levels (132/85 mmHg vs. 128/79 mmHg); as well as higher percentage of individuals with glucose metabolism abnormalities (85% vs. 63%) and lower frequency of pharmacotherapy (35% vs. 84%) including statin therapy (12% vs. 79%) and antihypertensive therapy (35% vs. 63%). The real-life data related to the baseline characteristics of the European MetS population that were collected in the TREMNIOS study indicate an urgent need to explore new preventative and therapeutical approaches for reducing cardiometabolic risks.

Our findings indicate that the feasibility of the 12-week TRE intervention with a self-selected 10-h EW (mostly “late TRE” pattern) in the European MetS population was satisfactory given that the adherence to TRE was 87% in the whole study group and reached 94% in the subgroup of patients who achieved the desirable TRE EW, i.e., EW ≤ 10 h/day (mean EW of 9.6 h). This observation is important, especially considering that there are only scarce data on the feasibility of TRE intervention in patients with MetS or MetS components [38,50,52,54]. Moreover, definitions of adherence to TRE and methods of capturing food intake were different in various studies [38,44,50,52,54]. Our findings are consistent with the results of the US study of subjects with MetS who underwent a 12-week TRE intervention with a 10-h EW according to a similar study protocol [38]. In that study, the adherence to TRE, defined as an intake of 95% of all calorie-containing ingestion events within a self-designated EW on the days recorded by the mCC app, was 93%. In the study by Kesztyüs et al. [50] including a European cohort of patients with MetS components who underwent a 12-week TRE intervention with self-selected 8–9 h EW, adherence to TRE defined as a proportion of days with fasting ≥15 h was 86%. Notably, obese sedentary or moderately active adults were compliant with a designated and significantly shorter (i.e., 4 h or 6 h) EW for ~6 days/week [44]. The findings of our study and a few other studies suggest that self-selecting EW during TRE may facilitate maintaining satisfactory adherence to TRE, particularly if the TRE intervention has been conducted over a long-term period [38,50]. Notably, in the study [38], ~63% of MetS patients were still somehow adherent to TRE in a period of 16 ± 4 months. However, a few TRE studies conducted in overweight or obese subjects reported lower adherence to TRE (~47–56%) despite self-selecting an 8–10 h EW and using the validated mCC app for recording food intake [43,52].

The use of a smartphone application to capture real-time data on a food intake is a unique tool to monitor the circadian rhythms of daily behavior and adherence to TRE intervention [25,37]. Our findings confirm a usefulness of a validated smartphone application such as the mCC app for monitoring TRE intervention. While the adherence to logging food intake decreased during TRE intervention compared to the baseline period, it seemed to be satisfactory during TRE, which might be associated with good compliance with TRE requirements. Specifically, the adherence to logging on the mCC app during TRE intervention was 70% in the whole study group and 74% in the group of patients who achieved the goal of mean TRE daily EW of ≤10 h. In the study of the US MetS population undergoing TRE [38], the adherence to logging meals on mCC app also decreased during the 12-week TRE intervention compared to baseline period; however, it was still high during TRE (i.e., 86%). Low adherence to logging meals during TRE intervention may indicate difficulties to comply with the TRE requirements [52]. In our study, the adherence to logging on the mCC app during TRE intervention in the group of patients who did not achieve the desirable reduction of EW during TRE was relatively low (i.e., 62%). In the study of Prasad et al. [52] of overweight and obese subjects with a prolonged daily eating period, a relatively low adherence to logging on the mCC app of 64% was accompanied by a low adherence to TRE (i.e., 47%). However, in the study of overweight subjects with daily EW ≥ 14 h [43], the adherence to TRE was relatively low (i.e., 56%) despite a satisfactory adherence to logging on the mCC app of 83%. Notably, a delay of the end time of daily EW during TRE did not correlate with either logging adherence or EW adherence in that study.

Based on our findings, the 12-week TRE intervention was effective in terms of reducing the duration of daily EW in the European population with the diagnosis of MetS and prolonged daily eating period. Specifically, mean daily EW was reduced by ~28% (i.e., by ~3.9 h) in the entire study group and by ~33% (i.e., by ~4.7 h) in the subgroup of patients who achieved the goal of mean daily EW ≤ 10 h during TRE. Our findings are consistent with the results of the study of US patients with MetS [38] (mean daily EW was reduced by ~29%, i.e., by ~4.3 h) and studies of overweight or obese US subjects (e.g., by ~35%, i.e., by ~5.3 h in [43] and by ~26%, i.e., by ~4.1 h in [52]).

The results of our study indicate that the 12-week TRE intervention with a self-selected EW (mostly “late TRE” pattern) resulted in favorable cardiometabolic effects, such as a significant decrease in BW, BMI, WC, hip circumference, systolic BP, and glycemic parameters, and improvements in wellbeing. We observed that a higher adherence to TRE and greater reduction in daily EW could be associated with substantial improvements in cardiometabolic outcomes. Specifically, a significant decrease in BW, BMI, WC, mean CGM fasting blood glucose level, liver function markers, sleepiness score, and depression score, as well as some improvements in hip circumference, FPG, HbA1c, and mean CGM 24-h blood glucose level were observed in individuals who had the adherence to TRE as high as 94% and achieved a substantial reduction in daily EW (by ~33%; i.e., ~4.7 h) with the mean EW during TRE of 9.6 h. In addition, we observed significant positive correlations between mean daily EW duration during TRE and post-TRE mean CGM fasting and 24-h blood glucose levels. However, favorable TRE effects were smaller (WC, FPG, and HbA1c) or not observed (BW, BMI, mean CGM blood glucose level, liver function markers, and sleepiness and depression scores) in the subgroup of patients characterized by adherence to TRE of 77%, reduction in daily EW by ~21% (i.e., ~2.9 h), and mean EW during TRE of 10.7 h. Nevertheless, it is worth emphasizing that some beneficial effects of TRE, such as a decrease in systolic BP, hip circumference, and atherogenic lipid levels, were observed in the subgroup of patients undergoing TRE though they did not achieve a desirable reduction in daily EW. Further clinical and basic research is needed to investigate mechanisms of cardiometabolic benefits caused by TRE, which may include antioxidative and anti-inflammatory actions, as well as a direct impact of TRE on circadian rhythm disruption [25,57].

A decrease in BW of ~2.3% (i.e., by ~2.4 kg), which was observed in our study after the 12-week TRE in the MetS patients who achieved the mean daily EW ≤ 10 h during TRE, is consistent with a decrease in BW of 2–4% post-8–10 h TRE reported by other studies of subjects with metabolic disorders undergoing TRE [25] including the US MetS population [38] (~3%, ~3 kg post 10-h TRE) and overweight or obese individuals (e.g., ~4% post 8-h TRE in [43]); ~2% post 10-h TRE in [52]; ~2% post 9-h TRE in [50]). A similar extent of BW reduction (~3%) was reported among sedentary or moderately active overweight or obese subjects after implementing TRE with a significantly shorter EW (i.e., 4 h and 6 h) compared to our study [44]. Also, a significant decrease in BW, albeit to smaller extent (~1–1.6%), was observed in some other TRE studies in the TRE group but the change was not significant compared to the control group [40,58]. However, it should be noted that in the study of Lowe et al. [58] including overweight or obese subjects undergoing 8-h TRE, most participants self-reported their weight changes using a Bluetooth weighing scale that was linked to a custom app. Moreover, no additional tools such as the mCC app were used to record food intake and monitor an adherence to TRE in that study. In the study of Philips et al. [40] of individuals with at least one component of MetS, only modest post-TRE reduction in BW might have resulted from implementing a 12-h EW that was longer compared to other human and animal studies which showed the benefits from a 4-10 h TRE intervention [26,38,43,44]. It should be also noted that we did not observe a decrease in BW in the subgroup of MetS patients who had lower adherence to TRE, smaller reduction in daily EW, and did not achieve a shortening in EW of ≤10 h/day.

Post-TRE reduction in daily caloric intake and number of eating occasions/day are among the factors that can affect TRE-related cardiometabolic effects, including a decrease in BW. In the US study of MetS patients who lost weight post TRE [38], a 9% decrease in mean daily caloric intake was found despite no recommendations to make changes in quality, quantity, or caloric content of diet during TRE [38]. Also, a 22% [43] to 50% [52] reduction in the number of eating events per day during TRE intervention were reported. In addition, a significant positive correlation between the percentage of weight loss and the number of ingestion events during TRE intervention was found [40]. In our study, while participants were not instructed to change their habits regarding physical activity or the content of diet, a decrease in a daily caloric intake (by ~16%) and the number of eating occasions (by ~10%) was observed. However, a decrease in daily caloric intake was not significant in the subgroup of patients with a mean TRE EW ≤ 10 h who lost weight. Notably, the post-TRE weight reduction observed in our study in MetS patients with a mean TRE EW of 9.6 h is comparable to the effects of both calorie restriction combined with exercise in subjects with glucose intolerance [59,60] and comprehensive intensive cardiac rehabilitation enhanced by low-calorie plant-based diet and psychosocial support in patients with CVD and numerous metabolic risk factors [61].

The post-TRE decrease in BW of ~2–3% could account for beneficial impact on abdominal adiposity indices, such as a 3% decrease in WC, which was observed in our study in participants with a mean TRE daily EW ≤ 10 h. A decrease in WC (by ~2–4%) was also found post TRE in other clinical studies of patients with MetS or MetS components [38,40] and abdominal obesity [43,50,52]. A decrease in WC in patients with metabolic disorders correlated with a change in BW and EW [38,43]. No change in VF and BF was found in our study, which was also observed in a few other TRE studies of patients with obesity and a high risk of T2D despite a post-TRE decrease in BW [45,46]. A greater reduction in EW duration during TRE than in our study and the use of dual-energy X-ray absorptiometry instead of bioelectrical impedance technology for measuring BF might be related to a post-TRE decrease in VF and BF that was observed in some other TRE studies of patients with metabolic disorders [38,43,44,50,62]. Muscle mass was maintained post TRE in the MetS subjects included in our study, which was not observed in other TRE studies of obese midlife subjects [43,58].

Our findings indicate a beneficial effect of TRE on systolic BP that decreased post TRE by ~5 mmHg (i.e., ~4%) and reached optimal level post TRE [23]. This effect was independent of the presence or absence of post-TRE weight loss and a degree of compliance to TRE requirements. TRE was shown to result in a reduction in BP in a few other TRE studies, including ones with obese subjects [45,52], a US population of patients with MetS [38], and obese adults with prediabetes, even in the absence of weight loss [54]. The 4% post-TRE decrease in systolic BP in our study is comparable to or even greater than that expected by weight loss through other means including multi-component comprehensive intensive cardiac rehabilitation [61,63]. The low rate of antihypertensive pharmacotherapy in our study compared to the study of Wilkinson and Manoogian et al. [38] including the US population with MetS (35% vs. 84%) could contribute to the smaller BP reduction in our study, especially that TRE can increase the efficacy of pharmacotherapy that also exhibits circadian rhythms [64]. However, no change in BP was observed in a few other TRE studies of obese adults and patients with MetS components, so further studies on the impact and mechanisms of TRE effects on BP are needed [40,44,58].

Based on our findings, 12 weeks of TRE with a self-selected 10-h EW resulted in significant improvements in glycemic parameters. Specifically, a 4% decrease in FPG (by ~4 mg/dL), a 4% decrease in HbA1c (by ~0.2%), and a 4% decrease in mean CGM fasting blood glucose level (by ~4 mg/dL) were observed. Moreover, FPG that was elevated at baseline reached the normal level (<100 mg/dL) post TRE. Notably, favorable post-TRE changes in FPG and HbA1c were observed in MetS patients undergoing TRE independently of the degree of EW reduction. However, improvements in the CGM blood glucose levels including a significant 6% decrease in mean CGM fasting blood glucose level (by ~5 mg/dL) as well as a trend toward lowering mean CGM 24-h blood glucose level were found only in the subgroup of patients with a mean TRE EW of ≤10 h/day. Moreover, significant positive correlations between the duration of TRE daily EW and post-TRE fasting and 24-h CGM blood glucose levels were found that may indicate an association between shortening daily EW and decreasing glucose levels post TRE. Regarding potential mechanisms of improving glycemic control post TRE intervention, it has been postulated that prolonged fasting during TRE may induce the metabolic switch, which occurs when changing from fed to fasted state [32,39]. The metabolic switch induces hepatocyte production of ketone bodies, increasing insulin sensitivity and decreasing fat accumulation. Similar trends, albeit without statistical significance, regarding post-TRE improvements in glycemic parameters were observed in the US study of patients with MetS undergoing TRE [38]; however, these beneficial TRE effects were more pronounced in patients with initially elevated FPG and/or HbA1c. In addition, while some improvements in FPG, CGM glucose levels, glucose tolerance, insulin levels, beta cell function, and insulin sensitivity and resistance were observed post TRE in some other studies of overweight or obese adults and obese subjects with a high risk of T2D (also independently of weight change and reducing caloric intake), significant improvements in glycemic parameters and insulin levels were found in subjects with elevated baseline FPG, HbA1c, and/or insulin levels [25,42,43,44,46,49,50,51,54,62]. Our results, indicating post-TRE benefits related to glycemic parameters in a MetS population, 85% of which had elevated baseline FPG and/or HbA1c, also suggest that patients with more impaired glucose metabolism may benefit more from TRE compared to lower-risk individuals. Further clinical and basic research is required to evaluate an impact and mechanisms of TRE on glucose metabolism [25,65].

Our findings related to the beneficial effects of a 12-week TRE intervention with a self-selected 10-h EW on wellbeing such as self-reported sleepiness and depression symptoms are consistent with the results of some other TRE studies, which reported post-TRE improvements in sleep duration and quality, subjective sense of energy level, feeling of wellbeing, and quality of life [32,33,38,45,47].

The TREMNIOS pilot clinical trial is a single-arm trial (pre–post TRE intervention) with no control group, which is a potential limitation [55]. This approach was chosen given that this trial is a precursor for a large-scale controlled trial in the future and has had limited funding that did not allow us to conduct a controlled pilot trial with a sufficient sample size of each group. Whereas the evaluation of the adherence to the TRE intervention does not require a control group, the interpretation of exploratory data on cardiometabolic parameters requires caution. The planned randomized controlled trial to be conducted after the TREMNIOS trial is expected to be free of this limitation.

The mCC app is designed to capture the timing of dietary intake but does not collect quantitate data on exact food portions or calorie estimates from the user. While calorie content was estimated from photo and text entries by participants, such data may be less precise than mechanistic studies where participants are supplied with specific foods. Also, one cannot exclude the potential effect of mCC app usage on participant behavior including dietary choices.

We believe that despite some limitations, the main value of our study is associated with targeting exclusively patients with diagnosed MetS and including a long duration of TRE intervention (12 weeks), a validated tool for recording food intake and sleep and monitoring adherence to TRE (mCC app), a comprehensive range of cardiometabolic outcomes, and the application of CGM for evaluating glucose control. To our knowledge, the TREMNIOS pilot clinical trial in Poland is the first study aimed at collecting feasibility and exploratory data on the effectiveness of TRE for improving cardiometabolic health and daily rhythms of behavior in a European adult population of patients with MetS and prolonged daily eating period.

Whereas multiple molecular mechanisms that can mediate the beneficial effects of TRE—such as improved insulin sensitivity, increased levels of fibroblast growth factor 21, reduced inflammation and oxidative stress, and enhanced cellular and molecular adaptive stress responses including improved mitochondrial function—have been considered, further basic and clinical research is needed to determine dominant pathway(s) of TRE’s effects and its ultimate potential for preventing and/or reversing cardiometabolic disorders [65].

## 5. Conclusions

The TREMNIOS pilot clinical trial provides exploratory data on the feasibility and effects of TRE intervention on cardiometabolic health and wellbeing in a middle-aged European population of patients with the diagnosis of MetS and prolonged daily eating period. Our findings indicate that the 12-week TRE with a self-selected 10-h EW was feasible; reduced daily EW; improved cardiometabolic outcomes, such as BW, BMI, WC, glucose levels, and systolic BP; and favorably affected wellbeing. Further shortening of daily EW was associated with greater benefits. The results of our study indicate that TRE can be an effective approach in subjects with MetS and high CV risk as a valuable component of non-pharmacological management or an addition to pharmacotherapy. We also demonstrate that the use of a validated smartphone application such as the mCC app is feasible and can be helpful for implementing TRE in the European MetS population. Further research is needed to investigate the mechanisms of TRE effects including an impact on circadian rhythm disruption.

The findings of this pilot clinical trial provide the basis for a planned large-scale randomized controlled trial to determine the efficacy and sustainability of TRE intervention for reducing long-term cardiometabolic risks, providing tools for sustained lifestyle changes and, ultimately, improving overall health in patients with MetS.

## Figures and Tables

**Table 1:** Eligibility criteria.

Inclusion Criteria
Age: 18–75 years BMI: ≥25 kg/m^2^ MetS defined as three or more of the following criteria: Elevated FPG ≥ 100 mg/dL Elevated waist circumference ≥ 102 cm in men, ≥88 cm in women Elevated fasting plasma TG ≥150 mg/dL (or drug treatment for elevated TG) Reduced HDL-C < 40 mg/dL for men, <50 mg/dL for women (or drug treatment for reduced HDL-C) Elevated BP, systolic BP ≥ 130 mm Hg and/or diastolic BP ≥ 85 mm Hg (or drug treatment for hypertension) Own a smartphone with Apple operating system (OS) or Android OS Average eating period of ≥14 h/day Habitual sleep duration of >6.5 h If patients are on cardiovascular medications (such as lipid-modifying drugs or anti-hypertensive drugs), no dose adjustments will be allowed during the study period
**Exclusion Criteria**
Diagnosis of diabetes Pregnant or lactating women Active smoking or illicit drug use or history of treatment for alcohol abuse Shift work Caregivers for dependent requiring nocturnal care Planned travel over one time zone during the study period History of a major adverse cardiovascular event within the past one year (acute coronary syndrome, percutaneous coronary intervention, coronary artery bypass graft surgery, hospitalization for congestive heart failure, stroke/transient ischemic attack) or current uncontrolled arrhythmia Uncontrolled medical conditions due to rheumatologic, hematologic, oncologic, infectious, gastrointestinal, psychiatric, nephrological, or endocrine diseases Known history of an eating disorder Currently enrolled in a weight loss or weight management program On a special or prescribed diet for other reasons (e.g., celiac disease) Current treatment with antidepressants, medication affecting glucose metabolism or appetite, or immunosuppression History of bariatric surgery A score of >16 on the Epworth Sleepiness Scale Depression determined by the Beck Depression Inventory-II (a score of >29) Failure to use the mCC app for documentation during a 2-week baseline period

Abbreviations: BMI—body mass index; BP—blood pressure; FPG—fasting plasma glucose; HDL-C—high-density lipoprotein cholesterol; mCC app—myCircadianClock application; MetS—metabolic syndrome; TG—triglycerides.

**Table 2:** Baseline demographic and clinical characteristics for the whole study group of patients with metabolic syndrome undergoing TRE and two patient subgroups depending on mean TRE daily eating window duration (>10 h or ≤10 h).

Variable	Whole Study Group (*n* = 26)	Subgroup with TRE Eating Window > 10 h (*n* = 11)	Subgroup with TRE Eating Window ≤ 10 h (*n* = 15)	*p*-Value between Subgroups with TRE Eating Window of >10 h or ≤10 h
Age (years)	45.4 (±12.6)	43.3 (±13.6)	47.0 (±12.0)	0.466
Gender (male/female) *n* (%)	10/16 (38.5/61.5)	5/6 (45.5/54.5)	5/10 (33.3/66.7)	0.689
White race *n* (%)	26 (100.0)	11 (100.0)	15 (100.0)	1.000
Body weight (kg)	103.0 (±19.4)	103.6 (±19.9)	102.5 (±19.7)	0.894
BMI (kg/m^2^)	34.5 (±5.1)	34.1 (±5.3)	34.8 (±5.2)	0.740
BMI ≥ 30/≥25–30 kg/m^2^ *n* (%)	20 (76.9)/6 (23.1)	9 (81.8)/2 (18.2)	11 (73.3/4 (26.7)	1.000
Waist circumference (cm)	113.8 (±13.2)	113.8 (±13.1)	113.9 (±13.7)	0.993
Hip circumference (cm)	120.2 (±11.1)	119.0 (±12.5)	121.1 (±10.3)	0.643
Systolic BP (mmHg)	132.4 (±12.1)	134.8 (±11.0)	130.7 (±12.9)	0.408
Diastolic BP (mmHg)	85.1 (±9.1)	83.5 (±7.9)	86.3 (±10.0)	0.446
Mean daily EW (h) ^a^	14.0 (±1.5)	13.6 (±1.4)	14.3 (±1.5)	0.204
Mean baseline adherence to logging on mCC app [%] ^b^	88.3 (±17.8)	83.5 (±21.7)	91.9 (±14.0)	0.816
MetS criteria (*n*/patient)	3.3 (±0.5)	3.2 (±0.6)	3.4 (±0.5)	1.000
Elevated FPG *n* (%)	20 (76.9)	8 (72.7)	12 (80.0)	1.000
Elevated waist circumference *n* %)	25 (96.1)	10 (90.9)	15 (100.0)	0.423
Elevated fasting plasma TG *n* (%)	14 (53.8)	8 (72.7)	6 (40.0)	0.130
Reduced HDL-C *n* (%)	9 (34.6)	2 (18.2)	7 (46.7)	0.394
Elevated systolic and/or diastolic BP *n* (%)	18 (69.2)	7 (63.6)	11 (73.3)	1.000
Family history of premature ASCVD *n* (%)	12 (46.1)	4 (36.4)	8 (53.3)	0.453
Regular aerobic exercise *n* (%)	12 (46.1)	5 (45.4)	7 (46.7)	1.000
Civil status: single/married *n* (%)	3 (11.5)/23 (88.5)	2 (18.2)/9(81.8)	1 (6.7)/14 (93.3)	0.556
Education: high school/higher education *n* (%)	10 (38.5)/16 (61.5)	6 (54.5)/5 (45.5)	4 (26.7)/11(73.3)	0.228
Employment: unemployed/employed/retired *n* (%)	2 (7.7)/21 (80.8)/3 (11.5)	1 (9.1)/9(81.8)/1 (9.1)	1 (6.7)/12 (80.0)/2 (13.3)	0.928
Domicile: village/city *n* (%)	4 (15.4)/22 (84.6)	1 (9.1)/10 (90.9)	3 (20.0)/12 (80.0)	1.000
Hemoglobin (g/dL)	14.4 (±1.5)	14.3 (±1.8)	14.5 (±1.2)	0.855
Leukocyte count (10^3^/µL)	6.7 (±1.9)	7.0 (±2.2)	6.4 (±1.5)	0.464
Creatinine (mg/dL)	0.80 (±0.14)	0.76 (±0.15)	0.83 (±0.13)	0.242
Uric acid (mg/dL)	5.9 (±1.2)	5.9 (±0.7)	5.9 (±1.5)	0.393
FPG (mg/dL)	102.0 (±8.6)	101.2 (±8.8)	102.7 (±8.7)	0.673
Glycated hemoglobin (%)	5.6 (±0.4)	5.7 (±0.5)	5.6 (±0.4)	0.723
Mean fasting blood glucose (mg/dL) ^c^	91.6 (±11.3)	94.4 (±9.3)	89.6 (±12.5)	0.294
Mean 24-h blood glucose (mg/dL) ^c^	96.4 (±9.6)	100.4 (±7.4)	93.5 (±10.2)	0.070
ALT (U/L)	37.3 (±19.9)	44.5 (±29.0)	33.7 (±13.8)	0.851
AST (U/L)	28.4 (±15.8)	32.4 (±22.3)	25.7 (±9.8)	1.000
TSH (mIU/L)	2.1 (±0.7)	2.0 (±0.8)	2.2 (±0.6)	0.433
TC (mg/dL)	200.3 (±36.5)	200.2 (±37.3)	200.4 (±37.2)	0.989
Non-HDL-C (mg/dL)	147.3 (±38.2)	147.8 (±39.5)	146.9 (±38.6)	0.953
HDL-C (mg/dL)	53.0 (±14.8)	52.4 (±7.5)	53.3 (±18.4)	0.874
LDL-C (mg/dL)	116.7 (±34.3)	115.9 (±39.0)	117.4 (±31.8)	0.916
TG (mg/dL)	153.5 (±50.7)	152.9 (±30.3)	153.9 (±63.6)	0.962
SCORE2 (%)	4.7 (±4.5)	4.2 (3.8)	5.1 (5.1)	0.597

^a^ Mean daily EW was calculated as a 2-week mean value using daily data recorded over the baseline period with the mCC app. ^b^ Mean adherence to logging on mCC app was calculated as a 2-week mean value using daily data recorded over the baseline period with the mCC app. ^c^ Mean glucose levels were calculated as a 2-week mean values using daily data recorded over the baseline period with CGM. Data represent mean values with standard deviation (in parenthesis). Abbreviations: ALT—alanine transaminase; ASCVD—atherosclerotic cardiovascular disease; AST—aspartate aminotransferase; BMI—body mass index; BP—blood pressure; CGM—continuous glucose monitor; EW—eating window; FPG—fasting plasma glucose; HDL-C—high-density lipoprotein cholesterol; LDL-C—low-density lipoprotein cholesterol; mCC app—myCircadianClock application; MetS—metabolic syndrome; non-HDL-C—non-high-density lipoprotein cholesterol; SCORE2—Systematic Coronary Risk Estimation2; TC—total cholesterol; TG—triglycerides; TRE—time-restricted eating; TSH—thyroid-stimulating hormone.

**Table 3:** Adherence to TRE and adherence to logging on the mCC app during TRE intervention for the whole study group of patients with metabolic syndrome undergoing TRE and two patient subgroups depending on mean TRE daily eating window duration (>10 h or ≤10 h).

Variable	Whole Study Group (*n* = 26)	Subgroup with TRE Eating Window > 10 h (*n* = 11)	Subgroup with TRE Eating Window ≤ 10 h (*n* = 15)	*p*-Value between Subgroups with TRE Eating Window of >10 h or ≤10 h
Duration of TRE intervention (days)	81.6 (±12.6)	80.5 (±17.9)	82.4 (±7.4)	0.896
Mean adherence to logging on mCC app during TRE intervention (%) ^a^	70.0 (±27.0)	62.0 (±27.6)	74.2 (±27.4)	0.152
Adherence to TRE intervention (%) ^b^	87.0 (±13.2)	77.3 (±14.2)	94.2 (±6.2)	**0.003**

^a^ Mean adherence to logging on mCC app was calculated as a 12-week mean value using daily data recorded over 12-week TRE intervention with the mCC app. ^b^ Adherence to TRE intervention was defined as a proportion of the total number of days recorded with the mCC app in which the participants satisfied a requirement of a 10 h eating window. Data represent mean values with standard deviation (in parenthesis). Abbreviations: mCC app—myCircadianClock application; TRE—time-restricted eating. Statistically significant differences are displayed in boldface font.

**Table 4:** Changes in eating window duration, adherence to logging on the mCC app, cardiometabolic outcomes, sleep, food intake, and questionnaires scores between the baseline period and TRE intervention for the whole study group of patients with metabolic syndrome undergoing TRE.

Parameter	Baseline (Mean (SD))	Post-TRE Intervention (Mean (SD))	Change between Baseline and TRE (Mean (SD))	Change between Baseline and TRE (%)	*p*-Value between Baseline and TRE
**Eating window**					
Mean daily EW (h)	14.0 (±1.5) ^a^	10.1 (±0.8) ^b^	−3.9 (1.7)	−27.9%	**0.0000009**
Mean adherence to logging on mCC app (%)	88.3 (±17.8) ^a^	70.0 (±27.0) ^b^	−18.3 (27.7)	−20.7%	**0.0002**
**Body weight and composition**					
Body weight (kg)	103.0 (±19.4)	101.3 (±19.1)	−1.7 (3.6)	−1.6%	**0.026**
BMI (kg/m^2^)	34.5 (±5.1)	34.0 (±5.1)	−0.5 (1.2)	−1.4%	**0.027**
Waist circumference (cm)	113.8 (±13.2)	111.3 (±13.5)	−2.5 (3.9)	−2.2%	**0.003**
Hip circumference (cm)	120.2 (±11.1)	118.1 (±11.2)	−2.1 (3.4)	−1.7%	**0.006**
Body fat (%)	37.1 (±8.8)	37.3 (±9.3)	+0.2 (2.5)	+0.5%	1.000
Visceral fat rating	12.2 (±4.5)	12.3 (±4.2)	+0.1 (2.2)	+0.8%	0.861
Muscle mass (kg)	61.6 (±13.4)	60.5 (±13.5)	−1.1 (3.7)	−1.8%	0.211
**Cardiovascular parameters**					
Systolic BP (mmHg)	132.4 (±12.1)	127.6 (±10.8)	−4.8 (9.0)	−3.6%	**0.012**
Diastolic BP (mmHg)	85.1 (±9.1)	84.1 (±8.2)	−1.0 (9.8)	−1.2%	0.620
Heart rate (bpm)	72.6 (±9.3)	75.0 (±11.4)	+2.4 (8.7)	+3.3%	0.540
SCORE2 (%)	4.7 (±4.5)	4.2 (3.9)	−0.5 (5.1)	−10.6%	**0.027**
**Glycemic parameters**					
FPG (mg/dL)	102.0 (±8.6)	98.2 (±8.7)	−3.8 (6.9)	−3.7%	**0.037**
Glycated hemoglobin (%)	5.6 (±0.4)	5.4 (±0.4)	−0.2 (0.4)	−3.6%	**0.011**
Mean fasting blood glucose (mg/dL)	91.6 (±11.3) ^c^	87.6 (±9.7) ^d^	−4.0 (6.1)	−4.4%	**0.002**
Mean 24 h blood glucose (mg/dL)	96.4 (±9.6) ^c^	94.6 (±10.8) ^d^	−1.8 (8.8)	−1.9%	0.313
**Lipids**					
TC (mg/dL)	200.3 (±36.5)	196.6 (±34.6)	−3.7 (28.4)	−1.8%	0.654
TG (mg/dL)	153.5 (±50.7)	140.8 (±37.0)	−12.7 (44.6)	−8.3%	0.166
HDL-C (mg/dL)	53.0 (±14.8)	52.5 (±15.3)	−0.5 (10.7)	−0.9%	0.689
Non-HDL-C (mg/dL)	147.3 (±38.2)	143.4 (±30.6)	−3.9% (28.2)	−2.6%	0.601
LDL-C (mg/dL)	116.7 (±34.3)	118.2 (±34.2)	+1.5 (26.5)	+1.3%	0.779
**Biochemical parameters**					
Creatinine (mg/dL)	0.80 (±0.14)	0.80 (±0.13)	0.00 (0.08)	0.0%	0.443
Uric acid (mg/dL)	5.9 (±1.2)	5.9 (±1.2)	0.0 (0.7)	0.0%	0.672
ALT (U/L)	37.3 (±19.9)	30.0 (±10.2)	−7.3 (26.2)	−19.6%	0.386
AST (U/L)	28.4 (±15.8)	22.8 (±9.7)	−5.6 (9.4)	−19.7%	**0.034**
Hemoglobin (g/dL)	14.4 (±1.5)	14.5 (±1.3)	+0.1 (0.6)	+0.7%	0.647
Hematocrit (%)	42.8 (±3.8)	43.1 (±3.5)	+0.3 (10.7)	+0.7%	0.312
Platelet count (10^3^/ µL)	287.9 (±66.2)	285.8 (±61.2)	−2.1 (30.7)	−0.7%	0.833
Leukocyte count (10^3^/ µL)	6.7 (±1.9)	6.4 (±1.8)	−0.2 (1.2)	−3.0%	0.233
Red blood cell count (mln/µL)	4.9 (±0.4)	4.9 (±0.4)	0.0 (0.2)	0.0%	0.771
**Sleep and questionnaires**					
Mean sleep duration (h)	7.6 (±0.7) ^a^	7.7 (±0.8) ^b^	+0.1 (0.8)	+1.3%	0.327
ESS (points)	7.7 (±4.0)	5.8 (±3.1)	−1.9 (3.2)	−24.7%	**0.043**
BDI-II (points)	7.6 (±3.9)	7.6 (±3.8)	0.0 (2.6)	0.0%	0.075
**Dietetic analysis**					
Mean daily caloric intake (cal)	1764.4 (±463.0)	1480.1 (±507.8)	−284.3 (395.8)	−16.1%	**0.002**
Mean number of eating occasions (*n*/day)	6.3 (±1.5)	5.7 (±1.7)	−0.6 (2.1)	−9.5%	0.069

^a^ Mean baseline value was calculated as a 2-week mean value using daily data recorded over the baseline period with the mCC app. ^b^ Mean post-TRE value was calculated as a 12-week mean value using daily data recorded over 12-week TRE intervention with the mCC app. ^c^ Mean baseline glucose levels were calculated as a 2-week mean values using daily data recorded over the baseline period with CGM. ^d^ Mean post-TRE glucose levels were calculated as a 2-week mean values using daily data recorded at the end of TRE intervention with CGM. Data represent mean values with standard deviation (in parenthesis). Abbreviations: ALT—alanine transaminase; AST—aspartate aminotransferase; BDI-II—Beck Depression Inventory-II; BMI—body mass index; BP—blood pressure; CGM—continuous glucose monitor; ESS—Epworth Sleepiness Scale; HDL-C—high-density lipoprotein cholesterol; LDL-C—low-density lipoprotein cholesterol; mCC app—myCircadianClock application; MetS—metabolic syndrome; Non-HDL-C—non-high-density lipoprotein cholesterol; SCORE2—Systematic Coronary Risk Estimation2; TC—total cholesterol; TG—triglycerides; TRE—time-restricted eating. Statistically significant differences are displayed in boldface font.

**Table 5:** Changes in eating window duration, adherence to logging on the mCC app, cardiometabolic outcomes, food intake, and questionnaires scores between the baseline period and TRE intervention within and between two patient subgroups depending on mean TRE daily eating window duration (>10 h or ≤10 h).

Variable	Subgroup with TRE Eating Window >10 h (*n* = 11)			Subgroup with TRE Eating Window ≤10 h (*n* = 15)			*p*-Value between Subgroups
	Baseline	TRE	*p*-Value	Baseline	TRE	*p*-Value	
**Eating window**							
Mean EW (h)	13.6 (±1.4) ^a^	10.7 (±0.6) ^b^	**0.00004**	14.3 (±1.5) ^a^	9.6 (±0.6) ^b^	**0.0003**	0.204/**0.00002**
Mean adherence to logging on mCC app (%)	83.5 (±21.7) ^a^	62.0 (±27.6) ^b^	**0.045**	91.9 (±14.0) ^a^	74.2 (±27.4) ^b^	**0.003**	0.140/0.152
**Body weight and composition**							
Body weight (kg)	103.6 (±19.9)	103.0 (±21.0)	0.413	102.5 (±19.7)	100.1 (±18.1)	**0.041**	0.894/0.705
BMI (kg/m^2^)	34.1 (±5.3)	33.9 (±5.5)	0.370	34.8 (±5.2)	34.0 (±5.0)	**0.045**	0.740/0.950
Waist circumference (cm)	113.8 (±13.1)	111.6 (±14.3)	0.072	113.9 (±13.7)	111.1 (±13.4)	**0.023**	0.993/0.927
Hip circumference (cm)	119.0 (±12.5)	117.0 (±12.9)	**0.016**	121.1 (±10.3)	119.0 (±10.1)	0.078	0.642/0.667
Body fat (%)	35.9 (±10.3)	36.0 (±10.6)	0.855	38.0 (±7.8)	38.2 (±8.5)	1.000	0.553/0.604
Visceral fat rating	11.9 (±4.6)	12.6 (±3.8)	0.468	12.4 (±4.5)	12.0 (±4.7)	0.138	0.788/0.714
Muscle mass (kg)	62.9 (±14.6)	63.3 (±15.3)	1.000	60.6 (±12.9)	58.4 (±12.2)	0.149	0.640/0.350
**Cardiovascular parameters**							
Systolic BP (mmHg)	134.7 (±11.0)	127.4 (±10.0)	**0.017**	130.7 (±12.9)	127.7 (±11.7)	0.239	0.408/0.933
Diastolic BP (mmHg)	83.5 (±7.9)	83.7 (±9.6)	0.904	86.3 (±10.0)	84.4 (±7.4)	0.536	0.446/0.841
Heart rate (bpm)	74.2 (±10.9)	77.1 (±12.6)	0.305	71.4 (±8.1)	73.4 (±10.7)	0.789	0.658/0.426
SCORE2 (%)	4.2 (±3.8)	3.6 (±3.7)	0.221	5.1 (±5.1)	4.6 (±4.1)	0.134	0.597/0.475
**Glycemic parameters**							
FPG (mg/dL)	101.2 (±8.8)	97.1 (±8.8)	0.076	102.7 (±8.7)	99.0 (±8.9)	0.068	0.673/0.594
Glycated hemoglobin (%)	5.7 (±0.5)	5.5 (±0.4)	0.090	5.6 (±0.4)	5.3 (±0.4)	0.061	0.723/0.280
Mean fasting blood glucose (CGM) (mg/dL)	94.4 (±9.3) ^c^	91.9 (±5.5) ^d^	0.231	89.6 (±12.5) ^c^	84.4 (±11.0) ^d^	**0.003**	0.294/0.051
Mean 24 h blood glucose (CGM) (mg/dL)	100.4 (±7.4) ^c^	101.5 (±6.8) ^d^	0.693	93.5 (±10.2) ^c^	89.5 (±10.4) ^d^	0.066	0.070/**0.003**
**Lipids**							
TC (mg/dL)	200.2 (±37.3)	180.1 (±29.8)	**0.032**	200.4 (±37.2)	208.6 (±33.8)	0.253	0.989/**0.035**
TG (mg/dL)	152.9 (±30.3)	143.4 (±28.3)	0.518	153.9 (±63.6)	138.7 (±43.6)	0.218	0.962/0.762
HDL-C (mg/dL)	52.4 (±7.5)	51.1 (±10.7)	0.730	53.3 (±18.4)	53.5 (±18.3)	0.302	0.874/0.755
Non-HDL-C (mg/dL)	147.8 (±39.5)	129.8 (±27.7)	0.079	146.9 (±38.6)	153.3 (±29.5)	0.345	0.953/0.051
LDL-C (mg/dL)	115.9 (±39.0)	103.7 (±33.0)	0.058	117.4 (±31.8)	128.9 (±32.1)	0.126	0.916/0.062
**Selected biochemical parameters**							
ALT (U/L)	44.5 (±29.0)	35.3 (±12.9)	0.617	33.7 (±13.8)	28.4 (±9.3)	0.077	0.851/0.250
AST (U/L)	32.4 (±22.3)	25.7 (±14.1)	1.000	25.7 (±9.8)	20.9 (±4.8)	**0.016**	1.000/1.000
**Sleep and questionnaires**							
Mean sleep duration (h)	7.7 (±0.6) ^a^	8.1 (±0.6) ^b^	0.164	7.5 (±0.7) ^a^	7.5 (±0.8) ^b^	0.961	0.535/0.053
ESS (points)	8.2 (±4.3)	8.1 (±3.4)	0.935	7.1 (±3.7)	5.2 (±2.7)	**0.041**	0.583/**0.047**
BDI-II (points)	7.4 (±4.0)	8.0 (±3.7)	0.450	7.0 (±3.5)	2.8 (±1.1)	**0.023**	0.842/**0.023**
**Dietetic analysis**							
Mean daily caloric intake (cal)	1744.5 (±563.5)	1352.7 (±446.7)	**0.023**	1777.1 (±408.5)	1562.0 (±543.3)	0.054	0.873/0.347
Mean number of eating occasions (*n*/day)	5.9 (±1.7)	6.0 (±2.5)	0.779	6.5 (±1.4)	5.6 (±1.4)	**0.045**	0.265/0.598

^a^ Mean baseline value was calculated as a 2-week mean value using daily data recorded over the baseline period with the mCC app. ^b^ Mean TRE value was calculated as a 12-week mean value using daily data recorded over 12-week TRE intervention with the mCC app. ^c^ Mean baseline glucose levels were calculated as a 2-week mean values using daily data recorded over the baseline period with CGM. ^d^ Mean post-TRE glucose levels were calculated as a 2-week mean values using daily data recorded at the end of TRE intervention with CGM. Data represent mean values with standard deviation (in parenthesis). Abbreviations: ALT—alanine transaminase; AST—aspartate aminotransferase; BDI-II—Beck Depression Inventory-II; BMI—body mass index; BP—blood pressure; CGM—Continuous Glucose Monitor; ESS—Epworth Sleepiness Scale; HDL-C—high-density lipoprotein cholesterol; LDL-C—low-density lipoprotein cholesterol; mCC app—myCircadianClock application; MetS—metabolic syndrome; Non-HDL-C—non-high-density lipoprotein cholesterol; TC—total cholesterol; TG—triglycerides; TRE—time-restricted eating. Statistically significant differences are displayed in boldface font.

## Data Availability

The data presented in this study are available on request from the corresponding author. The data are not publicly available due to privacy restrictions.
